# New Symptom of Pneumonia

**Published:** 1852-09

**Authors:** Wm. M. Boling

**Affiliations:** Montgomery, Ala.


From the American Journal of Medical Science.

*New Symptom of Pneumonia.* By Wm. M. Boling, M. D., of Montgomery, Ala.

I have frequently observed in pneumonia a symptom of which I do not remember to have seen mention made by any other, and which I have never noticed in any other disease. It consists in a deposition on the teeth, just along the margin of the gums, of a matter of different shades of color, from a light orange to a dull vermilion, forming a line about the sixteenth of an inch wide, and of a deeper tint at the gums, and paler as it recedes. Unlike the blue line said to be found in the margin of the gums in lead poisoning, and the line on the same part, of a deeper shade than the rest of the gum, noticed by Dr. Theophilus Thompson in phthisis, and mentioned in the *London Lancet*, for September, 1851. The appearance in question is seated on the teeth; from which, indeed, with care, it may be principally removed by wiping, though, occasionally, a somewhat durable stain remains upon the enamel.

In regard to the manner of its production, I am at a loss for an explanation, though it is probably an exudation from the margin of the gums. At first I thought it might be produced by the deposition of the coloring matter of the expectoration, but I have seen it in cases in which bloody matter was not expectorated; indeed, in a few cases of latent pneumonia, where there was neither cough nor expectoration; and, in one instance, I was led to suspect the presence of this form of the disease, which I ascertained with certainty by auscultation, by this symptom alone. Perhaps the miasmatic poisoning of the system may, in some way, lead to its development in pneumonia; for it is likely, that, if it were of as frequent occurrence in other localities as in this, it would have been noticed before. Still, I do not remember to have seen it in any of the forms of uncomplicated miasmatic fever.

I have made no note of the proportion of cases in which I have observed it, but I think, at least, in one-third or one-fourth. The cases in which it is present are generally severe, it being very rarely found in mild cases.

